# Expression of Concern for Woehlbier et al., “Analysis of Antibodies Directed Against Merozoite Surface Protein 1 of the Human Malaria Parasite *Plasmodium falciparum*”

**DOI:** 10.1128/iai.00543-24

**Published:** 2025-01-13

**Authors:** American Society for Microbiology

## EXPRESSION OF CONCERN

The American Society for Microbiology (ASM) and *Infection and Immunity* (IAI) are issuing this Expression of Concern regarding the following publication:

Woehlbier U, Epp C, Kauth CW, Lutz R, Long CA, Coulibaly B, Kouyaté B, Arevalo-Herrera M, Herrera S, Bujard H. 2006. Analysis of antibodies directed against merozoite surface protein 1 of the human malaria parasite *Plasmodium falciparum*. Infect Immun 74:1313–1322. https://doi.org/10.1128/iai.74.2.1313-1322.2006.

ASM and IAI were notified of serious concerns regarding this study. The Corporación Autónoma Regional del Valle del Cauca indicated in their investigative report, Resolución 0710 No. 0711 de 2024, the following violations:

- Not having the required permit to capture squirrel monkeys.
- Not having the required permit to experiment on monkeys.
- Not having the required permit to use animals or obtain any product from them.
- Committing “harm to wildlife.”

Additionally, Colombian authorities have closed down the facility (M. Wadman, Science, 2023, https://doi.org/10.1126/science.adi4928). Subsequently, the NIH defunded Caucaseco Scientific Research Center and removed it from its list of approved animal facilities (P. Jacobs, Science, 2023, https://doi.org/10.1126/science.adj6184).

ASM has formally requested that this paper and these concerns be reviewed as part of an ongoing “scientific misconduct investigation” by the Universidad del Valle and are awaiting their response. ASM is following its Publishing Ethics Policies and Procedures by publishing this Expression of Concern to raise awareness of the above-mentioned concerns; it will be updated accordingly pending the outcome of the investigation.

